# Dual Monoclonal Antibody Therapy for a Severe Asthma Patient

**DOI:** 10.3389/fphar.2020.587621

**Published:** 2020-09-30

**Authors:** Christian Domingo, Xavier Pomares, Anisi Morón, Ana Sogo

**Affiliations:** ^1^ Pulmonary Service, Corporació Sanitària Parc Taulí, Barcelona, Spain; ^2^ Department of Medicine, Universitat Autònoma de Barcelona (UAB), Barcelona, Spain; ^3^ Pharmacy Department, Corporació Sanitària Parc Tauli, Barcelona, Spain

**Keywords:** severe asthma, Th2 (type-2) immune responses, T2 immune responses, omalizumab, mepolizumab, combined therapy

## Abstract

**Introduction:**

Omalizumab, the first biological treatment for severe allergic bronchial asthma, has been on the market for more than a decade. Omalizumab was initially considered to be an IgE-blocking agent, and therefore, an inhibitor of the Th2 (allergic or adaptive) cascade. More recently, other monoclonal antibodies for severe eosinophilic asthma have become available, which exert an anti-eosinophilic effect basically by blocking IL5 or its receptor. These agents exert this effect regardless of the origin of the eosinophils (i.e., the adaptive or the innate immune system).

**Case study:**

An oral corticosteroid-dependent allergic asthma patient was treated with omalizumab. After a year of treatment, the improvement remained very limited and the medical team proposed discontinuation. However, the patient felt that her asthma had improved and she refused to give up the therapy, which continued for ten years. The mean accumulated oral corticosteroid dose per month during the last year was around 200 mg; despite this, the FEV_1_ was low, Since the patient had a high number of eosinophils in peripheral blood, she accepted a switch to mepolizumab when this agent became available. One year later, the clinical improvement was limited and severe symptoms of allergy reappeared, and a combination of monoclonal antiobodies (omalizumab and mepolizumab) was proposed.

**Results:**

After 24 months of dual therapy, a marked improvement in the FEV_1_ was observed, reaching the normal range, and the OC dose was reduced to 2.5 mg per day of prednisolone. No side effects were observed.

**Conclusions:**

In some severe allergic asthma patients with persistently high eosinophil counts in peripheral blood and who are considered non- or mild responders to anti-IgE and anti-IL5 administered individually, a combination of the two antibodies covering the entire T2 spectrum may be effective.

## Introduction

Until omalizumab was first marketed in 2006, the international guidelines considered oral corticosteroids (OCs) to be the final step in the treatment of severe asthma patients (Domingo et al., 2007). The use of certain immunosuppressive drugs was also proposed (Domingo et al., 2009; Domingo et al., 2011a) although they were not included in the guidelines. With the advent of biological treatments linked to the concept of precision medicine (Jameson and Longo, 2015), it became necessary to phenotype patients (Wenzel, 2012), and more recently to endotype them (Lötvall et al., 2011). As a result, the latest GINA update advises the use of monoclonal antibodies (mAbs) before chronic OCs and recommends mAbs as add-on therapy: omalizumab in allergic patients (IgE-mediated asthma), and anti-IL5 in eosinophilic patients (including anti-IL5r). Recently, dupilumab (an anti-IL4 and anti-IL13) was added to this group. Although these drugs have been shown to be highly efficacious (Humbert et al., 2005; Ortega et al., 2014) and real-life studies report an even greater effectiveness than randomized studies (Domingo et al., 2011b; Lugogo et al., 2016), not all patients respond favorably. Little is known about the consequences of switching from one family of mAbs to another, and there is no experience with mAb combinations. Here, we report the improvement in a patient with severe allergic asthma with a high eosinophil count treated with two concomitant mAbs.

## Case Study

A 55-year-old female was referred to our unit 14 years ago for management of her severe asthma. She had never smoked, had not worked for the last 10 years due to her disease (previously she had worked in a photography and printing shop), had no regular contact with animals, and her past clinical history did not reveal any relevant disease. Her asthma started at the age of 23 and was initially classified as allergic. She had been a patient at our hospital throughout her adult life, and the skin prick test using standardized products (ALK-Abelló) found in her hospital records showed allergy to house dust mites, *cupressus*, banana tree and dog epithelium. The patient was monitored by her general physician until she started to deteriorate. She was treated with a fixed dose combination of salmeterol 50 µg and fluticasone 500 µg bid, montelukast 10 mg per day and salbutamol on request. Since she had needed repeated bursts of OCs during the three years previous to her hospital visit, these drugs had been chronically prescribed. The year prior to starting treatment with omalizumab she had had five exacerbations requiring OCs. On referral to our unit, she reported dyspnea on mild exertion, frequent wheezing, and high consumption of rescue medication. She also reported some allergic comorbidities such as eye lachrymation and itching, rhinorrhea, and coryza. The new skin prick test using standardized antigens from the same company (ALK-Abelló) showed the same sensitization results. Total IgE value was 132 IU/ml. The peripheral blood eosinophil count was 400 (6.4%) and the FENO (Niox, Phadia^®^) score was 232 ppb.

The following anthropometric data were recorded: height 155 cm; weight: 72 Kg; body mass index (BMI): 29.9 kg/m^2^. Spirometry showed a severe lung obstruction with an FEV_1_ of 1.03 L (38% of the predicted figure) despite administration of 15–20 mg/day of prednisolone as add-on therapy. A computerized tomography of the paranasal sinuses ruled out nasal polyposis but confirmed some inflammation, and a chest CT scan showed air trapping and ruled out other lung diseases. Before starting the biological treatment, the adherence to inhalation therapy was confirmed by checking the electronic drug delivery of inhalers in pharmacies, while the inhalation technique was systematically checked at each visit by respiratory nurses.

The patient was administered sub-cutaneous (sc) omalizumab at a dose of 300 mg/month, in accordance with the dosing table (Domingo et al., 2007). After 16 weeks of treatment, she reported a mild improvement and the treatment was maintained. After a year of omalizumab, the improvement remained very limited (GETE: mild responder; asthma control test (ACT):12) and the medical team proposed discontinuation of the treatment. However, the patient felt that her asthma had improved and she refused to give up the treatment, which continued for ten years. During this period she did not suffer severe asthma exacerbations requiring hospital admission, her ACT ranged between 10–14, and she was unable to discontinue OCs. When mepolizumab became available, the medical team proposed a switch from omalizumab to this agent since the blood eosinophil count had remained repeatedly high (always above 400, and up to 1600 in one measurement). The patient agreed to the change, and a mild improvement was observed (ACT= 15) with reduction of the mean OC dose to 7.5-10 mg/day without significant changes in FEV_1_. After 14 months of a monthly sc mepolizumab dose of 100 mg, the clinical signs of allergy re-appeared. At that point, the medical team suggested that a combination of two mAbs administered concomitantly on the same day (omalizumab 300 mg/month plus mepolizumab 100 mg per month, both sc) might offer a synergistic benefit.

## Results

**Figure 1:**
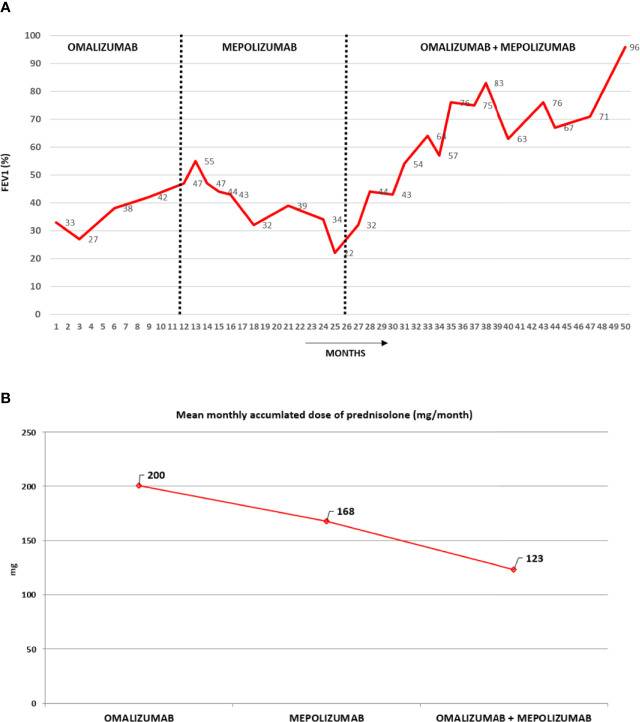
**(A)** The figure shows the changes in the FEV_1_ during three different periods. In the first period, the patient was receiving omalizumab, in the second mepolizumab, and in the third, mepolizumab and omalizumab. Note the marked improvement in the FEV_1_ during the dual mAb therapy. **(B)** The values of oral corticosteroid are the mean monthly accumulated intake during the treatment period of the last year of omalizumab alone, the 14 months on mepolizumab and the 24 months on omalizumab plus mepolizumab. A clear trend towards a decrease in oral corticosteroid consumption was observed. During the last three months, the daily OC dose was 2.5 mg/day.

After 24 months of dual biological therapy, the patient presented a notable improvement. At month 6, her ACT was 17 and ranged between 17 and 20 during the following six months. At the end of the first year, the FEV_1_ was normal (83% of the predicted value - **Figure 1A**) and although it showed some variability during the second year, airway obstructions (when they occurred) were always mild. At the end of the second year, the FEV_1_ value was 96%. Meantime, daily OC dose was reduced to and maintained at 2.5 mgr/day due to the risk of adrenal insufficiency (**Figure 1B**); no exacerbations occurred; GETE: good/excellent; ACT: maintained ≥20). The concomitant treatment with two mAbs was well tolerated. **Figure 1B** shows the evolution of OC consumption during the last three years of treatment.

## Discussion

Omalizumab was initially considered to be an IgE blocker, but it was soon observed that many immunological changes occurred after its administration; it was then redefined as an immunoregulatory drug acting mainly in the adaptive immune system (Domingo, 2014). The drug has been shown to be effective in allergic patients regardless of the eosinophil count (Hanania et al., 2013; Busse, 2018; Humbert et al., 2018), and achieves a significant reduction in tissue eosinophils (Djukanović et al., 2004). In the view of many authors (including our own group) patients with positive allergic tests are considered to have an allergic phenotype despite having high eosinophil counts, and are therefore placed on omalizumab (Blaiss et al., 2017; Bousquet et al., 2017; Domingo, 2017; Israel and Reddel, 2017). On occasions the improvement is very limited (Braunstahl et al., 2013) and the blood eosinophil count remains high. In these cases, when mepolizumab became available, a switch was made from one mAb family to another. Mepolizumab has been found to be effective in sensitized patients (Pelaia et al., 2020). However, in our case, the improvement with mepolizumab was very limited, and the re-appearance of allergic symptoms in the absence of the anti-IgE suggested that the Th2 cascade remained active. Eosinophils are effector cells which can be recruited from the adaptive immune system (Th2 profile) or from the innate immune system (a concept termed the “T2 profile”, which encompasses both adaptive and innate activity) (Israel and Reddel, 2017). Although in allergic patients the origin of the eosinophils is the activity of the allergic cascade itself (mast cells are the cells that release the highest amounts of IL5), the toxic effect of activated eosinophils can damage the bronchial epithelium and favor the release of alarmins (TSLP and IL33) that will activate type 2 innate lymphoid cells (ILC2) and natural killer T cells (NKT), which in turn will release IL5 and thus raise the eosinophil count (Israel and Reddel, 2017). This synergistic effect of the two arms of immunity included under the umbrella of the T2 profile can explain why the clinical response to one mAb (either omalizumab or mepolizumab) was limited and why the concomitant administration of the two drugs achieved a marked clinical improvement.

In 2017, a patient with allergic bronchopulmonary aspergillosis was treated with omalizumab and mepolizumab (Altman et al., 2017). This patient was receiving high doses of OCs (1-2 mg/Kg) due to the aspergillosis, but did not present improvement. Her allergic status was not well documented when omalizumab was started; the dose of this drug was changed during the follow-up and anti-fungal drugs were also concomitantly administered. The follow-up period was short and so no evidence of long-term benefit can be ascertained. To our knowledge, the report we present here is the first of a severe asthma patient correctly phenotyped as allergic, who had peripheral blood eosinophilia and was successfully treated with two concomitant mAbs without any other potentially confusing treatment and with a long follow-up period (two years) that demonstrated the persistent benefits of the combination.

## Conclusions

In cases in which some biological overlap occurs (Tran et al., 2016), the first step should be to prescribe the adequate drug for the corresponding phenotype; if this approach fails, a switch to another mAb should be made. In some selected patients who partially respond to either mAb individually, dual concomitant mAb administration should be considered.

## Data Availability Statement

The original contributions presented in the study are included in the article; further inquiries can be directed to the corresponding author.

## Ethics Statement

Ethical review and approval was not required for the study on human participants in accordance with the local legislation and institutional requirements. The patient provided their written informed consent to participate in this study. Written informed consent was obtained from the individual(s) for the publication of any potentially identifiable images or data included in this article.

## Author Contributions

CD, XP, and AS were the pulmonologists involved in the ambulatory care of the patient in her monthly visits at the hospital day unit of the severe asthma unit of the Hospital during these fourteen years. CD is the head of the severe asthma unit who made the diagnosis of severe asthma and indicated the biologic treatment. He is also the author who wrote the manuscript. AM was the responsible of accepting the combination of both drugs and was the responsible of the delivering and control the correct treatment with omalizumab and mepolizumab. All authors contributed to the article and approved the submitted version.

## Conflict of Interest

The authors declare that the research was conducted in the absence of any commercial or financial relationships that could be construed as a potential conflict of interest.
